# Adsorption Characteristics of Chloride Ions by Calcined Hydrotalcite and Its Influence on the Salt Corrosion Resistance of Asphalt Binder

**DOI:** 10.3390/ma19030587

**Published:** 2026-02-03

**Authors:** Jun Sheng, Yingxue Zou, Yuejing Lv, Dan Huang, Zenggang Zhao, Yuanlin Ding, Siyu Cheng, Jinxian Xiao

**Affiliations:** 1 Guangdong Changzda Road Maintenance Co., Ltd., Guangzhou 510897, China; 2 School of Automobile and Traffic Engineering, Wuhan University of Science and Technology, Wuhan 430081, China; 3 School of Civil Engineering, Central South University of Forestry & Technology, Changsha 410004, China

**Keywords:** calcination temperature, calcined layered double hydroxides, chloride ion, adsorption kinetics, asphalt binder, salt erosion resistance

## Abstract

This study aims to address the issue of asphalt pavement performance deterioration caused by chloride salt erosion. Layered double hydroxides (CLDHs) calcined at different temperatures, including 400 °C, 500 °C, and 600 °C, were used for the modification of asphalt binder. The structural evolution and chloride ion adsorption characteristics of CLDHs were analyzed. The adsorption kinetic behavior of CLDHs for chloride ions was investigated by combining adsorption kinetic experiments and electrochemical titration experiments. Through characterizing the interfacial adhesion performance between CLDH-modified asphalt binder and aggregates, the chemical composition of asphalt–ash binder before and after salt corrosion, and the leaching stability of organic substances in an environment with abundant chloride ions, the influence of CLDHs on the salt corrosion resistance of asphalt–ash binder was quantified. The results indicate that chloride adsorption by CLDHs is predominantly chemisorption-driven. With increasing calcination temperature, the chloride adsorption capacity of CLDHs gradually improved. In chloride-rich environments, CLDHs significantly enhanced the interfacial adhesion between asphalt binder and aggregates, particularly for coarse aggregates with a particle size of 9.5–13.2 mm. Furthermore, CLDHs effectively suppressed the formation of carbonyl and sulfoxide groups during salt corrosion and substantially decreased the leaching of organic components from asphalt binder. In summary, CLDHs can specifically enhance the salt corrosion resistance of asphalt binder, with the 600 °C-CLDHs demonstrating the most significant improvement, followed by the 400 °C-CLDHs, while the 500 °C-CLDHs performed the least effectively.

## 1. Introduction

The total length of the Chinese coastline exceeds 32,000 km, ranking eighth in the world. The total area of saline–alkaline land is over 100 million hectares, ranking third globally, accounting for approximately one-tenth of the total saline–alkaline land area in the world [1,2]. These regions are usually key areas for Chinese economic development, especially coastal cities. Salt from seawater is introduced into asphalt pavements by frequent rainfall, salt spray, and tidal phenomena in coastal areas, while salt within the soil is infiltrated into asphalt pavements through capillary action by rainwater and groundwater in saline–alkaline regions. Therefore, in these areas, salt is usually present in the service environment of asphalt pavements with water as the solvent. Water molecules and salt ions can penetrate the asphalt film, where physicochemical reactions such as oxidation, emulsification, dissolution, and migration of asphalt binder are triggered, inducing cohesive damage within the asphalt binder [3]. Additionally, the interface between the asphalt binder and aggregates can be infiltrated by water and salt, leading to the occurrence of adhesive damage at the interface [4]. Consequently, various distresses, including spalling, bleeding, potholing, and cracks, are induced in asphalt pavements. Driving safety is severely compromised, the service life of pavements is significantly shortened, and substantial economic losses as well as adverse social impacts are caused [5]. Thus, enhancing the durability of asphalt pavements in salt-rich environments has emerged as a critical scientific issue that urgently demands attention in the field of road engineering.

The approaches to enhancing the durability of asphalt pavements in salt-rich environments are primarily categorized into two types. The first involves improving the initial pavement performance of asphalt binder and asphalt mixtures through the optimization of material formulations and preparation processes [6]. Significant attention has been devoted to comprehensively enhancing the initial pavement performance of asphalt mixtures, with researchers worldwide focusing on aspects such as the modification of asphalt binder, additive application, and optimization of aggregate gradation, thereby enabling resistance to salt erosion [7]. However, several limitations are associated with this indirect salt corrosion resistance approach, including a lack of specificity, inadequate corrosion inhibition efficiency, imbalances in other performance metrics, and high costs.

In contrast, the second type of approach focuses on the intrinsic nature of salt corrosion. By leveraging the damage mechanism of asphalt binder induced by salt erosion, targeted modifications are made to material components and microstructures to directly improve the salt corrosion resistance of asphalt binder [4]. This anti-salination method based on the material’s salt erosion mechanism has been well established and applied in cement concrete. Because chloride ions account for the largest proportion of salt in natural environments and have the greatest impact on pavement materials, it is common to use materials that can fix chloride ions to reduce the harmful chloride ions in concrete [8]. This provides a new perspective for research on anti-salination methods based on the salt corrosion mechanism of asphalt mixtures. Commonly used materials include mineral powder, fly ash, layered double hydroxides (LDHs), and their calcined products (calcined layered double hydroxides, CLDHs) [9,10]. Among these, LDHs and CLDHs have also been widely utilized for immobilizing heavy metal anions and adsorbing chloride ions in wastewater [11,12]. The host layers of LDHs carry positive charges, while the hydroxyl and oxygen ions between the layers are negatively charged, resulting in electrostatic attraction. Interlayer anions and molecules are bonded through hydrogen bonds, and the layered spatial structure of hydrotalcites is formed by the stacking of these layers [13]. After LDHs are subjected to high-temperature calcination, their layered structure is destroyed, and the resulting products are all metal oxides known as CLDHs. Upon immersion in chloride salt solutions, metal hydration reactions occur, triggering the crystallization and reorganization of the material. During this process, ionized chloride ions in the solution are adsorbed, and the layered structure is regenerated. This constitutes the mechanism underlying the adsorption of chloride ions by leveraging the structural memory effect of LDHs [14].

Despite the significant potential of CLDHs in chloride ion immobilization, the existing research has predominantly focused on their application in cement-based materials, leaving a distinct gap in their exploration for application in the modification of asphalt binder. Firstly, there is a lack of systematic investigation into the regulatory effects of calcination temperature on the structural evolution and chloride ion adsorption capacity of CLDHs. Notably, changes in key characteristics, such as specific surface area and active sites with calcination temperature, directly influence salt corrosion resistance. Secondly, limited research has been conducted on the salt corrosion resistance of CLDH-modified asphalt binder.

Therefore, the study focuses on the adsorption characteristics of chloride ions by CLDHs and their influence on the salt corrosion resistance of asphalt binder. Structural characterization and chloride ion adsorption kinetic tests were carried out to clarify the structural evolution law of CLDHs under different calcination temperatures and the intrinsic mechanism of their chloride ion adsorption behavior. Moreover, modified water immersion tests, Fourier transform infrared (FTIR) spectroscopy, and total organic carbon (TOC) measurements were combined to conduct a systematic evaluation regarding the effects of CLDHs on the asphalt–aggregate interfacial adhesion, the oxidative degradation degree of asphalt chemical components, and the dissolution and migration characteristics of asphalt binder in chloride-rich environments. This study is intended to provide a theoretical basis and technical support for the development of high-performance salt-corrosion-resistant asphalt binders that are applicable to chloride-rich service environments.

## 2. Methodology: Materials and Experiments

### 2.1. Materials

#### 2.1.1. Asphalt Binder

Base asphalt binder with 60/80 penetration grade (70A) produced by PetroChina (Beijing, China) was adopted, and the basic technical indicators are presented in Table 1.

#### 2.1.2. LDHs

**Figure 1:**
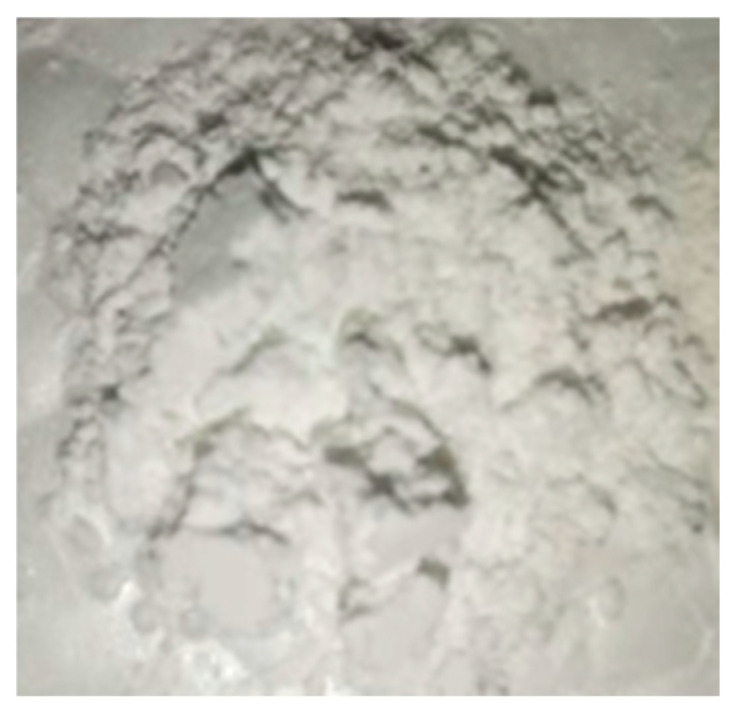
Morphology of LDHs.

The magnesium–aluminum layered double hydroxide (Mg-Al-LDH) containing interlayer carbonate anions, produced by Beijing Taikelai Chemical Co., Ltd. (Beijing, China), was adopted, and the technical information of this LDH is presented in Table 2. The morphology of LDHs is shown in Figure 1.

#### 2.1.3. Aggregates

Natural basalt aggregates with particle sizes ranging from 9.5 to 13.2 mm, widely used in asphalt pavements, were selected in this study, and the chemical composition is presented in Table 3. They sourced from Wuhan Jiuhua Co., Ltd. (Wuhan, China). With a silica (SiO_2_) content of 45.27%, basalt belongs to mafic aggregates, exhibiting advantages such as strong adhesion to asphalt binder, high compressive strength, and excellent corrosion resistance—thus being extensively applied in pavement engineering [22].

#### 2.1.4. NaCl

NaCl is the predominant salt present in the environments of coastal areas and saline-soil regions. Therefore, NaCl form Wuhan Jiangcheng Chemical Co., Ltd. (Wuhan, China) was selected as the salt for indoor saline-environment simulation tests, and the technical properties are shown in Table 4. To avoid interference from soluble calcium salts, magnesium salts, and other impurities that may exist in domestic water and impact the results of salt corrosion simulation tests, deionized water subjected to purification treatment was used throughout the study for all the experiments.

### 2.2. Research Methodology

**Figure 2:**
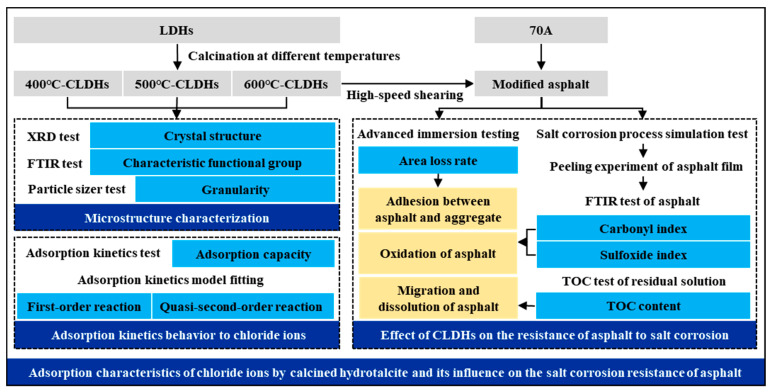
Research schema.

The research schema is illustrated in Figure 2. Structural characterization and adsorption kinetic experiments were conducted to reveal the structural evolution of CLDHs with varying calcination temperatures and the intrinsic characteristics of their chloride ion adsorption behavior. Combined with modified water immersion tests, FTIR spectroscopy, and total organic carbon (TOC) test, a comprehensive investigation was performed to evaluate the effects of CLDHs on three key aspects of asphalt binder in salt-rich environments: the adhesion between asphalt binder and aggregates, the oxidation degree of chemical compositions, and the dissolution and migration characteristics.

### 2.3. Preparation of CLDHs

To investigate the chloride ion adsorption behavior of calcined hydrotalcite, three calcination temperatures (400 °C, 500 °C, and 600 °C) were selected, and the calcination process was conducted as follows [23]. Firstly, the hydrotalcite powder was placed in a vacuum-drying oven and dried at 105 °C for 12 h to completely remove physically adsorbed water, thereby avoiding interference of moisture on crystal structure changes during calcination. The calcination experimental steps are detailed below:

Step i: A box-type high-temperature resistance furnace (Manufacturer: Jinan Luhe Instrument Co., Ltd., Jinan, China; model: SX2-4-10; temperature control accuracy: ±1 °C) was used as the calcination equipment. The dried hydrotalcite powder was evenly spread in corundum crucibles (loading thickness ≤ 2 cm) to ensure uniform heating.

Step ii: The temperature was raised from room temperature to the target temperature (400 °C, 500 °C, or 600 °C) at a heating rate of 5 °C/min, which prevents abrupt removal of interlayer water in hydrotalcite caused by rapid heating, thereby avoiding particle fragmentation or structural collapse.

Step iii: Upon reaching the target temperature, isothermal calcination was maintained for 5 h. During this stage, interlayer water molecules (removed at approximately 200 °C) and interlayer anions (e.g., CO_3_^2−^, decomposed at approximately 400 °C) in hydrotalcite are gradually removed, and lattice reconstruction of layered metal hydroxides is promoted to form amorphous or semi-crystalline composite metal oxides (Mg-Al-O solid solution).

Step iv: The heating power was turned off, and the temperature inside the furnace was allowed to cool naturally to room temperature (cooling rate: approximately 10 °C/min) to avoid internal stress generation in CLDH particles due to rapid cooling. The prepared CLDHs were thus obtained. The above steps were repeated to produce CLDHs at calcination temperatures of 400 °C, 500 °C, and 600 °C, respectively.

### 2.4. Preparation of CLDH-Modified Asphalt Binder

To ensure the uniform dispersion of CLDHs in asphalt binder, a high-speed shear emulsifier was employed for processing. The asphalt and CLDHs with a weight of 5 wt% of the asphalt were subjected to high-speed shear dispersion at 130 °C and a rotational speed of 3000 revolutions per minute for 30 min. CLDH-modified asphalt binders with different calcination temperatures were prepared separately, including 400 °C-CLDH-modified asphalt binder, 500 °C-CLDH-modified asphalt binder, and 600 °C-CLDH-modified asphalt binder.

### 2.5. Experiments

#### 2.5.1. X-Ray Diffraction (XRD) Test

XRD tests were performed on LDH and CLDH samples to characterize their crystal structure characteristics. An XRD (Bruker Corporation, Karlsruhe, Germany, D8 Advance) was used, with a Cu Kα target as the X-ray source (emitted X-ray wavelength: 0.15406 nm). During the test, the scanning range was set to 5–80° (2θ) at a scanning speed of 0.02°/s.

#### 2.5.2. Chloride Ion Adsorption Kinetic (CIAK) Test

Adsorption kinetic tests were conducted to characterize the chloride ion adsorption behavior of CLDHs, with LDHs set as the blank control group. The tests enabled the determination of the time required to reach adsorption equilibrium, which serves as a crucial reference for the effective action time in practical applications. Meanwhile, kinetic parameters obtained by fitting the test data facilitate the analysis of the adsorption mechanism [24]. Briefly, 0.5 g of CLDH samples was weighed and placed in stoppered glass bottles containing 100 mL of 300 mmol/L chloride ion solution, which was prepared by dissolving analytical-grade NaCl in deionized water. The bottles were then placed on a magnetic stirrer, and agitation was conducted at 25 ± 1 °C with a rotational speed of 2400 r/min to ensure sufficient contact between CLDHs and the chloride ion solution for adsorption reactions. At predetermined time intervals, namely 10 min, 20 min, 40 min, 100 min, 200 min, 400 min, 1080 min, and 1440 min, 5 mL of solution samples was collected from each bottle. The collected samples were immediately centrifuged at 1500 r/min for 5 min to separate CLDH particles, yielding clear filtrates. The residual chloride ion concentration in the filtrates was determined by potentiometric titration (Potentiometric titrator, Shanghai He Gong Scientific Instrument Co., Ltd., CT-1PLUS, Shanghai, China). Based on the change in chloride ion concentration before and after adsorption, the adsorption capacity of CLDHs for chloride ions was calculated using Equation (1):

(1) $$q_{t}\;=\;\frac{(C_{0}-C_{t})\;\times \;V}{m}$$

where *q_t_* denotes the adsorption capacity for chloride ions at time (mg/g), *V* represents the volume of the solution (L), and *m* is the mass of CLDHs (g).

#### 2.5.3. Modified Water Immersion (MWI) Test in Saline Solution

Adhesion failure is the most prominent manifestation of salt corrosion damage in asphalt mixtures, and it is one of the fundamental causes of asphalt pavement cracking. To more intuitively compare the effect of CLDHs on the interfacial adhesion performance between asphalt binder and coarse aggregates in a saline environment, a MWI test was conducted using a 10% NaCl solution. First, 100 g of coarse aggregates and 5.5 g of uncorroded CLDH-modified asphalt binder were thoroughly mixed at 135 °C. The asphalt binder-coated coarse aggregates were then immersed in NaCl solutions of different concentrations and soaked at 80 °C for 30 min. After the MWI test, Image-Pro Plus 6.0 software was used to process the surface images of the aggregates. Initially, the images were subjected to 8-bit processing to improve quality, followed by binarization processing to clearly identify the peeling areas of asphalt binder on the aggregate surface. Through these processing steps, the ratio of the peeling areas of asphalt binder to the total surface area of the aggregates could be accurately calculated, and this ratio was defined as the peeling rate.

#### 2.5.4. Salt Corrosion Process Simulation (SCPS) Test

**Figure 3:**
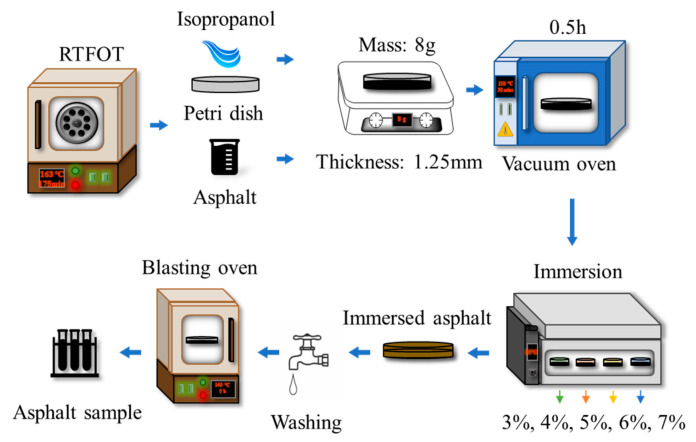
Flow chart of saline-environment simulation test.

In this study, an indoor saline environment was simulated by incorporating the salinity characteristics of the natural environment in coastal areas and saline-soil regions. Salt spray and seawater splash in coastal areas result in a salinity of up to 3% on asphalt pavements [25]. The salinity in the pavement environment of saline-soil regions primarily ranges from 0 to 3% [26]. However, humidity in the natural environment varies with seasons and temperature changes, leading to extreme salinity environments on local pavements [27]. NaCl is the most abundant salt in the natural environment, with a solubility in water of 37.3 g/100 mL at 60 °C [28]. Typically, a 10% NaCl concentration is used in accelerated salt corrosion tests in laboratories [29]. As is well known, pavement temperatures can reach 60 °C in most cases during summer. Therefore, a 10 wt% NaCl solution was prepared by dissolving solid NaCl in deionized water, and immersion tests were conducted on asphalt binder at 60 °C to simulate the saline environment encountered by asphalt pavement during service in high-temperature and rainy summer weather. The specific process is illustrated in Figure 3.

First, thermal–oxidative aging tests were performed on the asphalt binder samples using the Rolling Thin Film Oven Test (RTFOT) to simulate the thermal–oxidative aging process of asphalt binder during pavement transportation and paving. Petri dishes with a diameter of 90 mm were cleaned with isopropyl alcohol to reduce the risk of microbial growth on the inner walls. After drying, 8 g of molten CLDH-modified asphalt binder after RTFOT aging was poured into the Petri dishes, which were then placed in a vacuum-drying oven at 130 °C for 0.5 h. After cooling, asphalt binder films with a thickness of 1.25 mm were obtained. Subsequently, 40 mL of deionized water and 10% NaCl solution were, respectively, poured into the Petri dishes to completely submerge the asphalt binder films in the NaCl solution. The asphalt binder samples were immersed at 60 °C for 10 days. After the 10-day immersion test at 60 °C, the samples were processed in two sequential steps to obtain two types of test specimens: salt-corroded asphalt binder samples and residual-solution samples containing asphalt binder-derived substances. The detailed processing procedures are as follows: (1) The remaining NaCl solution was collected, and the asphalt binder films in the Petri dishes were slowly rinsed with deionized water for 5 min to remove salts attached to the film surfaces as well as substances dissolved and migrated from the asphalt binder. The aqueous solution used for rinsing the asphalt binder and the remaining NaCl solution were collectively collected and named NaCl solution containing asphalt binder residues (referred to as residual solution for short). Due to the low concentration of organic substances dissolved and migrated from the asphalt binder in the residual solution, carbon tetrachloride was selected as the extractant to extract these organic substances, thereby improving data reliability. First, the residual solution was poured into a separating funnel, 10 mL of carbon tetrachloride was added, and the funnel was shaken to ensure uniform mixing. It was then allowed to stand vertically for 5 min until the solution was completely stratified. The purified residual solution was collected from the bottom outlet to obtain the sample to be tested. (2) The rinsed asphalt binder films were placed in a blast oven at 40 °C for 24 h to remove surface moisture, yielding salt-corroded asphalt binder as the sample to be tested. To collect sufficient asphalt binder samples for testing, four parallel immersion tests were set up.

#### 2.5.5. Peeling Test for Asphalt Binder

**Figure 4:**
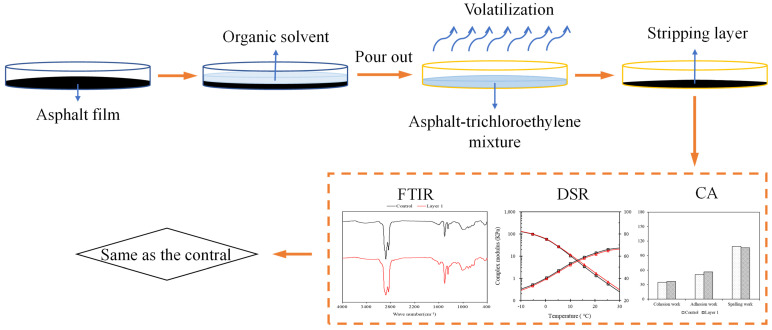
Flow chart of peeling test for asphalt binder.

To avoid the influence of uneroded asphalt binder in the inner layer on the test results, a peeling test was conducted to obtain surface asphalt binder by peeling the asphalt binder film. The specific operation steps are illustrated in Figure 4. First, 5 mL of trichloroethylene, an organic solvent, was poured into the Petri dish to dissolve the surface of the asphalt binder film. After 11 s, the upper mixed solution of asphalt binder and trichloroethylene was poured into a clean container. The container was placed in a fume hood for 72 h to allow complete evaporation of trichloroethylene, yielding the samples to be tested. FTIR tests, Dynamic Shear Rheometer (DSR) tests, and Contact Angle (CA) tests were performed to compare the infrared spectra of base asphalt binder before and after peeling with trichloroethylene. The results indicate that the peeling process had no significant effect on the chemical composition of asphalt binder, verifying the feasibility of this peeling method. The mass of the stripped asphalt binder layer was weighed, and the thickness of the stripped asphalt binder layer was calculated using Equation (2). It was determined that the calculated thickness of the asphalt binder film obtained after dissolution with trichloroethylene for 11 s was approximately 26 μm.

(2) $$T=\frac{m}{\pi r^{2}\rho }$$

where *T*, *m*, and *ρ* denote, respectively, the thickness, mass, and density of the asphalt binder film, and *r* represents the radius of the Petri dish.

#### 2.5.6. FTIR Test

FTIR (Nicolet 6700, Thermo Fisher Scientific, Waltham, MA, USA) test was used to characterize the changes in characteristic functional groups of CLDH-modified asphalt binder before and after salt corrosion, aiming to investigate the effect of CLDHs on the oxidation reaction of asphalt binder in chloride-rich environments. Carbon disulfide was selected as the organic solvent to prepare a 5 wt% asphalt solution. First, two drops of the asphalt solution were placed on potassium bromide (KBr) pellets and exposed to a warm light for 2 min to ensure complete evaporation of carbon disulfide prior to FTIR testing [30]. The scanning wavenumber range was set to 4000–400 cm^−1^ with 64 scans to guarantee the accuracy and reliability of the test results.

#### 2.5.7. TOC Test

A TOC analyzer (Multi-N/C2100S, Analytik Jena GmbH+Co. KG, Jena, German) was employed to determine the TOC content in the purified residual solution, enabling quantitative analysis of the content changes in water-soluble asphalt components and stripped asphalt components. The measurement range was 0–1000 mg/L, the injection volume was 50–500 μL, the detection limit was ≤0.5 mg/L, and the repeatability was ≤3%.

## 3. Results and Analysis

### 3.1. Microstructural Characterization of CLDHs

#### 3.1.1. Thermodynamic Property of CLDHs

**Figure 5:**
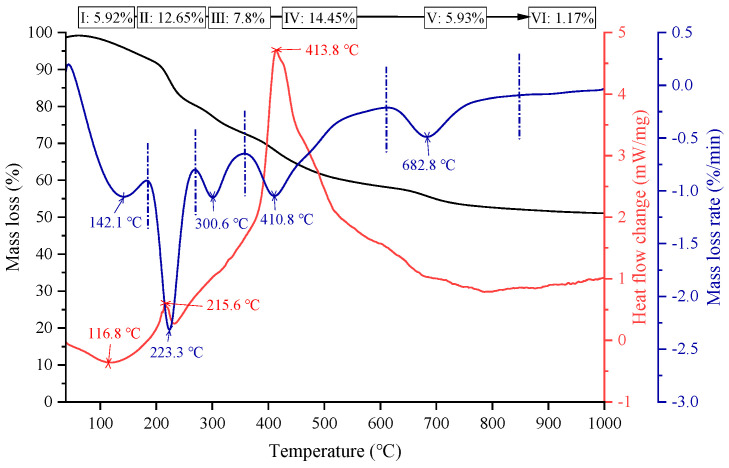
TG–DTA curves of LDHs.

This study aims to synthesize CLDHs with efficient chloride ion adsorption capacity. The calcination temperature must satisfy two key criteria: the complete decomposition of LDHs into highly active amorphous mixed metal oxides (MMOs) while avoiding excessive crystallization of MMOs as crystallization reduces reconstruction and adsorption capacity. Therefore, thermogravimetric analysis was performed on LDHs to study their thermodynamic properties, and the results are presented in Figure 5, which shows the curves of mass loss (TG), mass loss rate peaks (DTG), and heat flow change peaks (DSC) as a function of temperature. Based on the curves, the mass loss process of LDHs can be divided into six stages [31].

Stage I: The DTG peak at 116.8 °C and DTA peak at 142.1 °C correspond to the desorption of surface-adsorbed water, with a mass loss of 5.92%.

Stage II: The DTG peak at 215.6 °C and DTA peak at 223.3 °C are attributed to the removal of interlayer crystalline water, resulting in a mass loss of 12.65%.

Stage III: The DSC peak at 300.6 °C represents the maximum heat flow change point for the initial dehydration of layer hydroxyl groups and pre-decomposition of interlayer anions, which is dominated by endothermic reactions corresponding to preliminary chemical structure changes.

Stage IV: The DTG peak at 413.8 °C and DTA peak at 410.8 °C correspond to the core decomposition stage. Complete dehydration and condensation of layer hydroxyl groups (forming amorphous MMOs) and full decomposition/removal of interlayer anions (e.g., CO_3_^2−^→CO_2_↑) occur during this stage, which is a critical interval for thorough chemical structure reconstruction, with a mass loss of 14.45%.

Stage V: The DSC peak at 682.8 °C is the maximum heat flow change point for the removal of residual components and adjustment of the amorphous phase (endothermic peak, corresponding to the deep removal of residual hydroxyl groups/anions).

Stage VI: No significant DTG/DSC peaks are observed, and the curve tends to flatten, indicating that the amorphous MMOs are converted into stable crystalline phases (e.g., MgAl_2_O_4_ spinel). Only a minimal amount of impurities are removed, and the structure enters a thermally stable range.

In summary, below 400 °C, LDHs decompose incompletely, and residual hydroxyl groups/anions weaken the reconstruction activity of MMOs. Within the range of 400–600 °C, LDHs can be fully converted into amorphous MMOs with a high specific surface area and abundant active sites, enabling efficient Cl^−^ adsorption and CLDH reconstruction. Above 600 °C, MMOs are prone to excessive crystallization (e.g., spinel phase formation), leading to a significant decline in reconstruction and Cl^−^ adsorption performance. Thus, the optimal calcination temperature range is determined to be 400–600 °C.

#### 3.1.2. Crystal Structure of CLDHs

**Figure 6:**
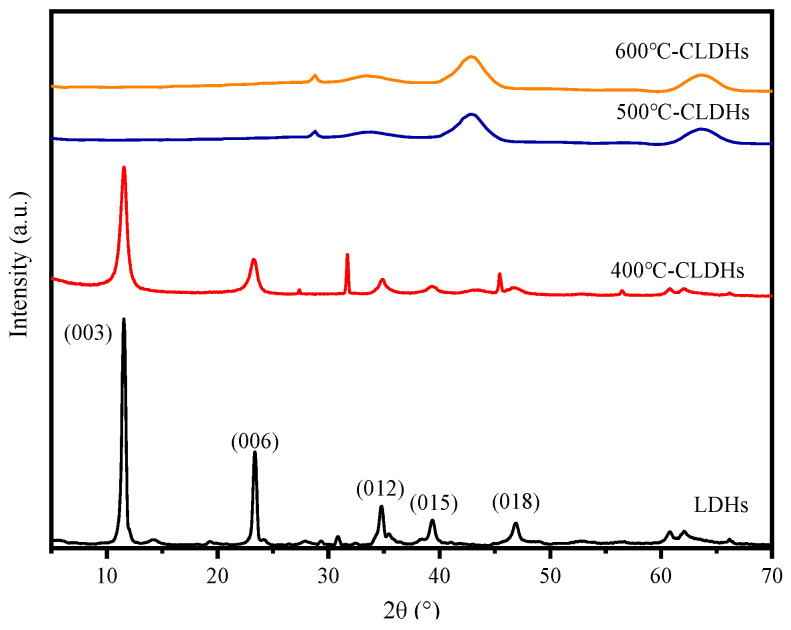
XRD pictures of LDHs and CLDHs.

The XRD patterns of LDHs and CLDHs are presented in Figure 6. The diffraction pattern of LDHs exhibits sharp and intense diffraction peaks at (003), (006), and (012) planes, along with characteristic peaks of (015) and (018) planes. These are typical characteristic peaks of LDHs, indicating a highly ordered layered structure, excellent crystallinity, and the absence of oxide phases [32]. For CLDHs calcined at 400 °C, the layered characteristic peaks remain clearly visible. Combined with the results in Figure 5, LDHs begin to decompose into oxide phases at 400 °C, suggesting that the layered structure is not fully decomposed. For CLDHs calcined at 500 °C, the layered characteristic peaks basically disappear without distinct layered crystal plane signals, and only characteristic diffraction peaks of magnesium–aluminum mixed oxides are observed. The characteristic peaks of CLDHs calcined at 600 °C are essentially consistent with those calcined at 500 °C, indicating that LDHs have been basically calcined into magnesium–aluminum mixed oxides at 500 °C.

#### 3.1.3. Characteristic Functional Groups of CLDHs

**Figure 7:**
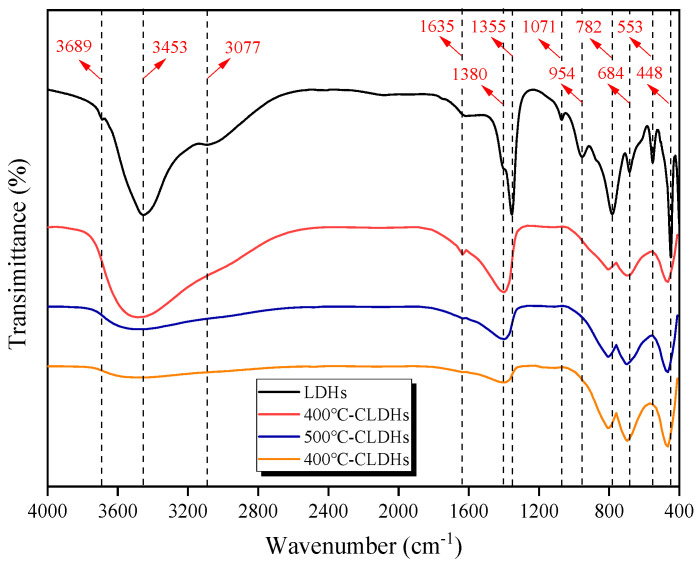
FTIR spectra of LDHs and CLDHs.

FTIR spectroscopy is an effective method for characterizing the functional group composition and structural evolution of CLDHs, with the corresponding FTIR spectra presented in Figure 7. The results indicate that the characteristic peaks of CLDHs calcined at different temperatures are essentially identical. An analysis of specific peaks in the FTIR spectra reveals 12 characteristic absorption bands within the wavenumber range of 4000–400 cm^−1^, with the detailed analysis provided in Table 5. Eight absorption peaks in the range of 4000–900 cm^−1^ correspond to the functional groups of layer hydroxyl groups, interlayer water hydroxyl groups, and interlayer CO_3_^2−^, while four absorption peaks in the range of 900–400 cm^−1^ are attributed to the functional groups of metal oxides. As the calcination temperature increases, the absorption peaks between 4000 and 900 cm^−1^ gradually weaken, whereas the intensity of characteristic peaks between 900 and 400 cm^−1^ gradually enhances, and the peak shapes become sharper. The phenomenon is attributed to the removal of physically adsorbed interlayer water, the gradual dehydration of layer hydroxyl groups, and the thermal decomposition of interlayer CO_3_^2−^, which escapes as CO_2_ during calcination. The layered structure of LDHs gradually collapses during the calcination process, and the metal ions in the layers recombine via M-O bonds to form composite metal oxides such as MgO and γ-Al_2_O_3_. This process corresponds to the phase transformation of CLDHs into metal oxides [32].

#### 3.1.4. Particle Size of CLDHs

Table 6 presents data on the specific surface area (SSA) and volume-average particle size of LDHs and CLDHs calcined at different temperatures. The SSA of LDHs is 2.08 m^2^/g with a volume-average particle size of 5.078 μm. After calcination at 400 °C, the SSA of CLDHs increases to 2.234 m^2^/g, while the particle size decreases to 4.740 μm. Combined with the results in Figure 5, LDHs lose interlayer anions and water molecules during the first stage of calcination at this temperature. The removal of interlayer guests induces the initial dissociation of the layered structure, and the formed micropores expand the exposed interface of the material, thereby increasing the SSA and reducing the particle size. When the calcination temperature is increased to 500 °C, the SSA of CLDHs decreases to 1.960 m^2^/g. As indicated in Figure 5, the crystalline phase of LDHs disappears at this temperature, and amorphous products are formed. The weak agglomeration effect of the amorphous products weakens the contribution of the porous structure to the surface area, leading to a temporary decrease in SSA and a slight increase in particle size. As the calcination temperature rises to 600 °C, the SSA of CLDHs rebounds to 2.994 m^2^/g. According to Figure 5, the formation of a large number of small pores in the layers at high temperatures triggers structural reconstruction. The abundant porous channels formed in the layers significantly expand the effective interface of the material, further increasing the SSA and resulting in further refinement of the particles.

### 3.2. Chloride Ion Adsorption Kinetic Behavior of CLDHs

To investigate the crystal structure characteristics and chloride ion adsorption behavior of CLDHs, XRD analysis, adsorption capacity testing, and kinetic model fitting were conducted. A comparison of the chloride ion adsorption behavior of CLDHs calcined at different temperatures was also performed.

#### 3.2.1. Relationship Between Chloride Ion Adsorption Capacity of CLDHs and Time

**Figure 8:**
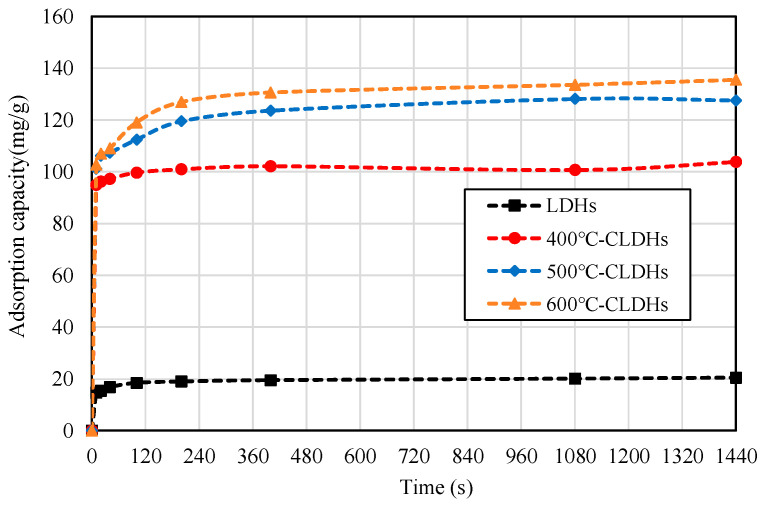
Chloride ion adsorption capacity of CLDHs at different times.

The residual chloride ion concentration in the filtrate at different time points was determined by potentiometric titration, and the corresponding adsorption capacity was calculated. The results are presented in Figure 8. It can be observed that the chloride ion adsorption capacity of both LDHs and CLDHs continuously increases with the extension of adsorption time. For LDHs, the adsorption capacity increases rapidly within the first 10 min and reaches equilibrium at approximately 100 min, after which no significant increase in adsorption capacity is observed. This phenomenon is attributed to the limited adsorption sites on the surface and between the layers of LDHs. For 400 °C-CLDHs, the chloride ion adsorption pattern is similar to that of LDHs, but the adsorption capacity is much higher, indicating that CLDHs exhibit a significant advantage in chloride ion adsorption. For 500 °C-CLDHs and 600 °C-CLDHs, the adsorption capacity increases rapidly within the first 10 min, followed by a slow increase from 10 to 400 min, and equilibrium is achieved at approximately 400 min. The adsorption patterns of these two types of CLDHs are similar.

#### 3.2.2. Kinetic Model Fitting for Chloride Ion Adsorption by CLDHs

To further investigate the chloride ion adsorption mechanism of CLDHs, the experimental data were fitted with the pseudo-first-order kinetic equation and pseudo-second-order kinetic equation, respectively [33]. Both are important tools for studying adsorption phenomena and evaluating adsorption performance. The pseudo-first-order kinetic model assumes that the adsorption rate is related to the residual adsorbate concentration in the solution, and it is often used for preliminary analysis of the adsorption process. A high fitting degree indicates that the adsorption process is predominantly controlled by diffusion. The pseudo-second-order kinetic model considers the chemical adsorption process. It assumes that the adsorption rate is determined by the chemical reaction at the active sites on the adsorbent surface, emphasizing the chemical interaction between the adsorbate and the adsorbent during adsorption. A good fit of the experimental data to the pseudo-second-order kinetic model implies that chemical adsorption plays a dominant role in the entire adsorption process. The pseudo-first-order kinetic equation is shown in Equation (3), which can be transformed into Equation (4). The pseudo-second-order kinetic equation is expressed as Equation (5):

(3) $$ln(q_{e}-q_{t})\;=\;lnq_{e}-k_{1}t$$

(4) $$q_{t}=q_{e}(1-e^{-k_{1}t})$$

(5) $$\frac{t}{q_{t}}=\frac{1}{k_{2}q_{e}^{2}}+\frac{t}{q_{e}}$$

where *q_e_* denotes the equilibrium adsorption capacity (mg/g), *k*_1_ is the pseudo-first-order kinetic rate constant (min^−1^), and *t* represents the adsorption time (min); *k*_2_ is the pseudo-second-order kinetic rate constant (g/(mg·min)), which can be obtained by linear fitting of the relationship between $\frac{t}{q_{e}}\;$ and *t* to determine the fitting parameters of the pseudo-second-order kinetic model.

**Figure 9:**
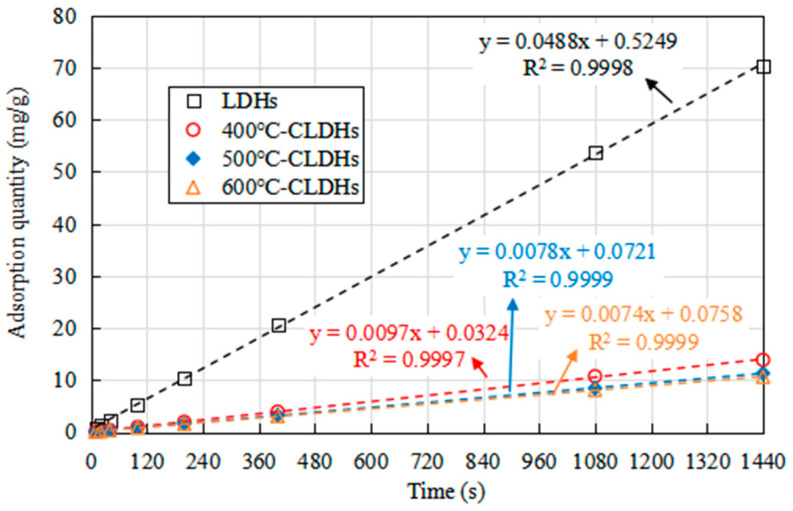
Pseudo-second-order kinetic fitting model for chloride ion adsorption by CLDHs.

The fitting results of the pseudo-first-order kinetic model are shown by the dashed lines in Figure 9, while those of the pseudo-second-order kinetic model are presented in Figure 8, with the corresponding fitting parameters listed in Table 7. The results indicate that the equilibrium adsorption capacities of LDHs, 400 °C-CLDHs, 500 °C-CLDHs, and 600 °C-CLDHs are 20.4918 mg/g, 103.0928 mg/g, 128.2051 mg/g, and 135.1351 mg/g, respectively. The correlation coefficient R^2^ values obtained from the pseudo-second-order kinetic model are higher than those from the pseudo-first-order model, and all exceed 0.999. This suggests that chemical adsorption may be the key factor determining and controlling the adsorption process.

Combined with the results in Figure 5, LDHs begin to decompose at 400 °C, and amorphous MMOs are formed through dehydration condensation of the layered structure, resulting in insufficient chemical active sites. At 500 °C, LDHs are basically completely decomposed, forming abundant MMOs with a high density of active sites. At 600 °C, the decomposition degree of LDHs is higher than that at 500 °C, accompanied by a larger specific surface area, more abundant porous structures, and increased chemical active sites. Consequently, 600 °C-CLDHs exhibit the maximum equilibrium adsorption capacity.

### 3.3. Effect of CLDHs on the Salt Corrosion Resistance of Asphalt Binder

To clarify the regulatory mechanism of CLDHs on the salt corrosion resistance of asphalt binder, this section focuses on analyzing the changes in interfacial adhesion, chemical composition, and material migration behavior of asphalt binder under a saline environment after modification with CLDHs calcined at different temperatures. For a comprehensive comparison, uncalcined LDHs were introduced as a control group, leveraging their intrinsic layered structure to form a physical barrier against chloride ions, thereby providing a reference for evaluating the unique effect of CLDHs.

#### 3.3.1. Adhesion Performance of CLDH-Modified Asphalt Binder in Chloride-Rich Environments

**Figure 10:**
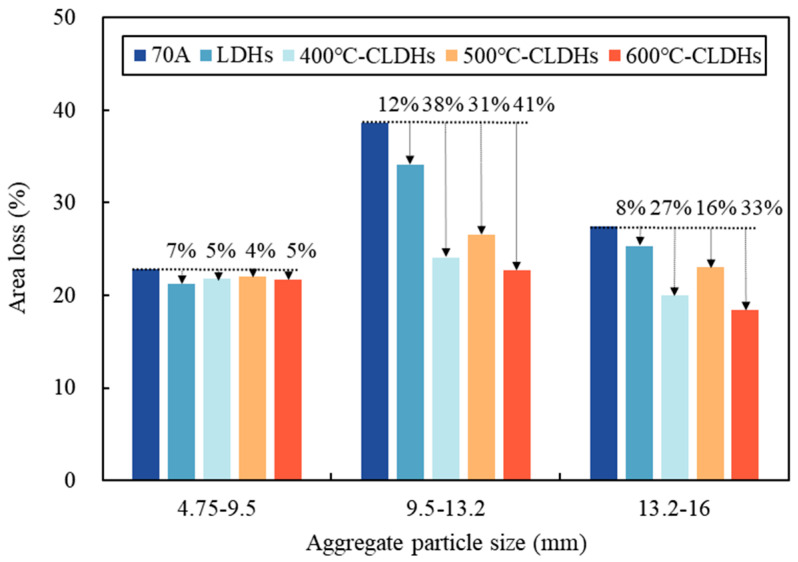
Area loss of modified asphalt binder and aggregates in saline environment.

Aggregates of different particle sizes were coated with uncorroded CLDH-modified asphalt binder and then subjected to a water immersion test in a saline solution. The results are presented in Figure 10. The peeling rate between asphalt binder and fine aggregates (4.75–9.5 mm) is relatively low, which may be attributed to the high specific surface area of fine aggregates providing sufficient interfacial contact area and bonding sites for asphalt binder. LDHs and CLDHs calcined at different temperatures slightly reduce the peeling rate, with no significant effect. Compared with fine aggregates (4.75–9.5 mm), the peeling rate between asphalt binder and coarse aggregates (9.5–13.2 mm) is higher, possibly due to the slightly smaller specific surface area of 9.5–13.2 mm aggregates. The incorporation of LDHs, 400 °C-CLDHs, 500 °C-CLDHs, and 600 °C-CLDHs reduces the peeling rate by 12%, 38%, 31%, and 41%, respectively, with the order of influence magnitude being LDHs < 500 °C-CLDHs < 400 °C-CLDHs < 600 °C-CLDHs. The ability of LDHs to reduce the peeling rate may be due to the fact that the layered structure of LDHs can physically block the penetration of water and salt ions into the asphalt–aggregate interface to a certain extent. As indicated in Table 7, the chloride ion adsorption capacity of CLDHs follows the order 400 °C-CLDHs < 500 °C-CLDHs ≤ 600 °C-CLDHs. According to Table 6, the specific surface area ranks as 500 °C-CLDHs < 400 °C-CLDHs < 600 °C-CLDHs. Further, 500 °C-CLDHs are in the transition stage from crystalline phase disappearance to amorphous phase formation, with a reduction in surface-active sites due to amorphous agglomeration. In contrast, 400 °C-CLDHs retain part of the layered structure, which exhibits a water-blocking effect, and the removal of interlayer guests exposes more surface-active sites. These active sites can form stronger chemical bonds with the acidic components of asphalt binder and the silica–alumina groups of aggregates, resulting in better interfacial bonding stability than the 500 °C-CLDH system. Therefore, the peeling rate of 400 °C-CLDH-modified asphalt binder is lower than that of 500 °C-CLDH-modified asphalt binder. Moreover, 600 °C-CLDHs have been completely converted into MMOs, which exhibit high chemical stability. Their specific surface area is significantly increased to 2.994 m^2^/g, with well-developed porous structures and abundant surface-active sites. The composite metal oxides can form coordination bonds/ionic bonds with the acidic components of asphalt binder and chemical bonds with aggregates (silicate-based) through interfacial hydroxyl groups, resulting in the highest interfacial bonding strength. Additionally, 600 °C-CLDHs possess the strongest chloride ion adsorption capacity. Consequently, they exhibit the lowest peeling rate in the saline environment.

#### 3.3.2. Characteristic Functional Group Indices of CLDH-Modified Asphalt Binder After Salt Corrosion

Salt corrosion induces chemical reactions, such as oxidation, dissolution, and migration, in asphalt binder [34]. The aging products of asphalt binder mainly contain functional groups, such as carbonyl and sulfoxide groups, and changes in the absorption peaks of these two groups are commonly used as indicators to analyze the effect of salt corrosion on asphalt binder. Therefore, FTIR tests were performed on asphalt samples before and after salt corrosion. The 2000–650 cm^−1^ range was selected as the reference to quantify the changes in two characteristic functional groups: carbonyl groups centered at approximately 1700 cm^−1^ and sulfoxide groups centered at approximately 1030 cm^−1^ [35]. The peak area ratios of the characteristic functional groups were calculated using Equations (6) and (7) to obtain the carbonyl index (*I_C_*_=*O*_) and sulfoxide index (*I_S_*_=*O*_) [36].

(6) $$I_{C=O}={\;A}_{1700}/\sum A_{2000\text{\textasciitilde{}}650}$$

(7) $$I_{S=O}={\;A}_{1031}/\sum A_{2000\text{\textasciitilde{}}650}$$

where $A_{1700{\mathrm{cm}}^{-1}}$ represents the absorption peak area of carbonyl groups centered at approximately 1700 cm^−1^, $A_{1030{\mathrm{cm}}^{-1}}$ denotes the absorption peak area of sulfoxide groups centered at approximately 1030 cm^−1^, and $\sum A_{2000\text{\textasciitilde{}}650}$ is the sum of the peak areas of different characteristic peaks within the wavenumber range of 2000–650 cm^−1^.

**Figure 11:**
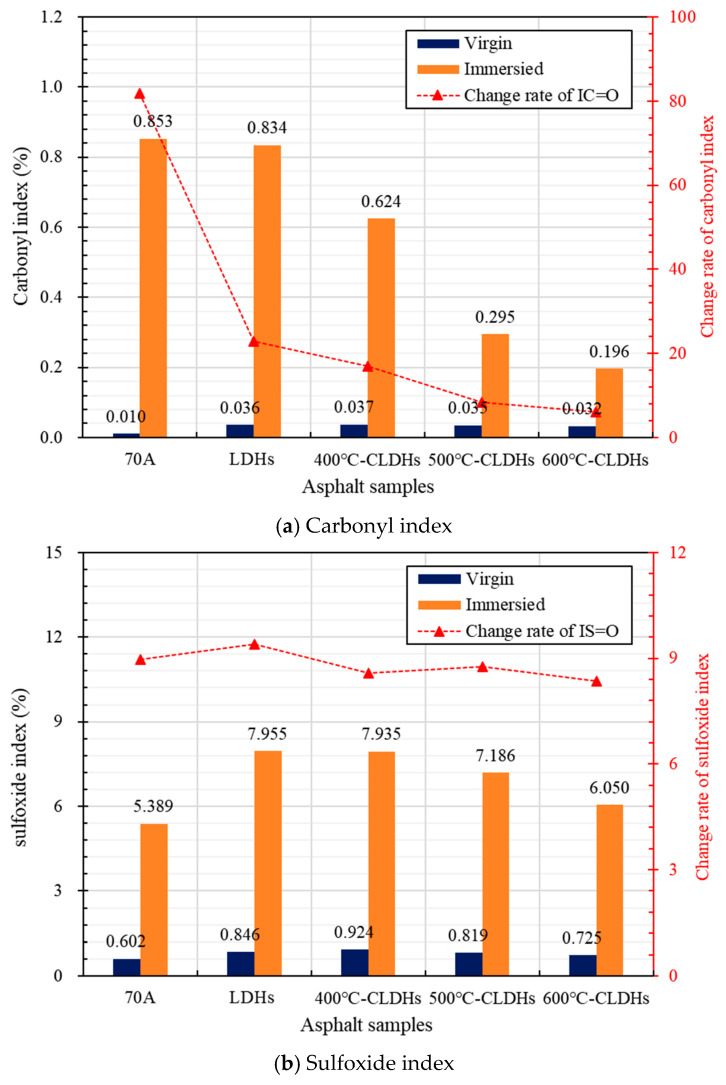
Indices of characteristic functional groups of asphalt samples before and after salt corrosion.

The calculated characteristic functional group indices of asphalt binder after salt corrosion are presented in Figure 11. From Figure 11a, it can be observed that, before immersion, the *I_C_*_=*O*_ of all the modified asphalt binders is slightly higher than that of 70A. This is attributed to the short-term thermal–oxidative aging of asphalt binder during the modification process, which generates a small amount of carbonyl groups. Before and after immersion, the order of the change rate of the *I_C_*_=*O*_ of asphalt samples is 70A > LDHs > 400 °C-CLDHs > 500 °C-CLDHs > 600 °C-CLDHs. This indicates that the incorporation of the three types of CLDHs can significantly reduce the formation of carbonyl-containing reaction products in asphalt binder induced by salt corrosion. The underlying mechanism may be that the layered structure of LDHs provides physical shielding against oxygen and water, 400 °C-CLDHs retain part of the layered structure while possessing a certain number of active sites and chloride ion adsorption capacity, and 500 °C-CLDHs and 600 °C-CLDHs exhibit stronger chloride ion adsorption capacity. Further, 600 °C-CLDHs exert the most significant effect on asphalt binder. Compared with 70A, the change rate of the *I_C_*_=*O*_ of 600 °C-CLDH-modified asphalt binder before and after salt corrosion decreases by 92.6%. Similarly, as shown in Figure 11b, the incorporation of CLDHs can slightly reduce the formation of sulfoxide-containing reaction products in asphalt binder caused by salt corrosion, and 600 °C-CLDHs demonstrate the most notable effect, with the change rate of *I_S_*_=*O*_ decreasing by 6.9% compared with 70A. Therefore, the incorporation of CLDHs can enhance the oxidation resistance of asphalt binder in a saline environment, and 600 °C-CLDHs exhibit optimal performance.

#### 3.3.3. Total Organic Carbon Content in Residual Aqueous Solution After Salt Corrosion

**Figure 12:**
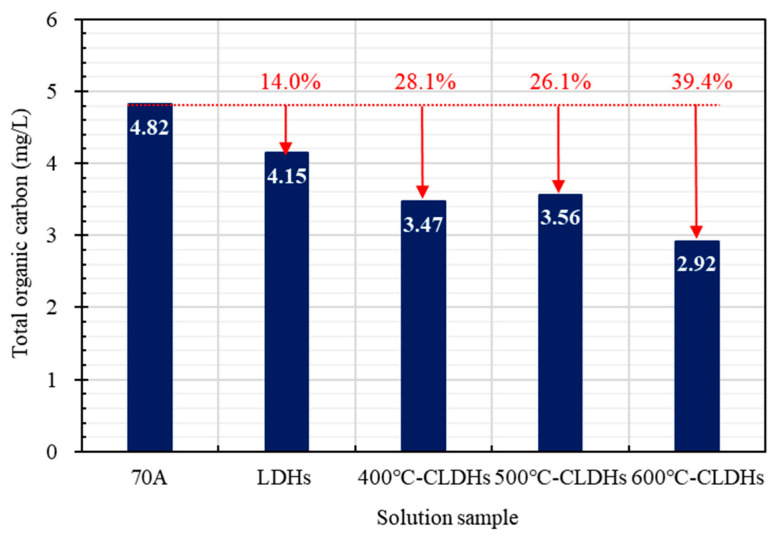
TOC content of the residual solution after immersion.

To quantify the substances dissolved and migrated from asphalt binder, the TOC content of the residual solution was characterized, with the results presented in Figure 12. The incorporation of LDHs, 400 °C-CLDHs, 500 °C-CLDHs, and 600 °C-CLDHs reduced the TOC content in the residual solution by 14.0%, 28.1%, 26.1%, and 39.4%, respectively. All of these materials effectively inhibited the dissolution and migration of asphalt binder in the saline solution. The intact layered structure of LDHs can form a complex physical barrier within asphalt binder, slightly retarding the leaching of organic components. Indeed, 400 °C-CLDHs contain both partial layered structures and metal oxides, exerting dual effects of physically blocking the leaching of components and chemically adsorbing easily leachable substances. Most 500 °C-CLDHs exhibit an amorphous structure but tend to agglomerate. Thus, their inhibitory effect on dissolution and migration is comparable to that of 400 °C-CLDHs. Further, 600 °C-CLDHs are mostly composed of MMOs with the largest specific surface area. They efficiently capture leached organic components through the dual effects of physical interception and chemical adsorption. Meanwhile, the chemical coordination bonds formed between the composite metal oxides and asphalt binder enhance intermolecular binding forces, further inhibiting the dissociation of organic components. Consequently, the TOC content is minimized, and 600 °C-CLDHs achieve the optimal leaching inhibition effect.

## 4. Summary and Discussion

This study combined the structural evolution and chloride ion adsorption characteristics of CLDHs calcined at different temperatures to analyze the chloride ion adsorption kinetic behavior of CLDHs. By characterizing the interfacial adhesion performance between CLDH-modified asphalt binder and aggregates in a chloride-rich environment, the chemical composition of asphalt binder before and after salt corrosion, and the organic leaching stability, the effect of CLDHs on the salt corrosion resistance of asphalt binder was quantified. The key experimental results are summarized as follows:

- The structure and physicochemical properties of CLDHs evolve with the calcination temperature gradient. At 400 °C, CLDHs retain a residual layered structure; at 500 °C, they transform into an amorphous structure; and, at 600 °C, they form MgO/γ-Al_2_O_3_ composite metal oxides. The specific surface area follows the order 500 °C-CLDHs < 400 °C-CLDHs < 600 °C-CLDHs, while the volume-average particle size shows the opposite trend.
- The chloride ion adsorption behavior of CLDHs conforms to the pseudo-second-order adsorption kinetic model (R^2^ > 0.999), indicating that chemical adsorption dominates the adsorption process. The calculated equilibrium chloride ion adsorption capacities of 400 °C-CLDHs, 500 °C-CLDHs, and 600 °C-CLDHs are 103.0928 mg/g, 128.2051 mg/g, and 135.1351 mg/g, respectively. The 600 °C-CLDHs exhibit optimal chloride ion adsorption efficiency due to their larger specific surface area and abundant active sites.
- CLDHs can enhance the adhesion performance between asphalt binder and aggregates of different particle sizes in a chloride-rich environment, with the most significant effect on coarse aggregates (9.5–13.2 mm). The incorporation of 400 °C-CLDHs, 500 °C-CLDHs, and 600 °C-CLDHs reduces the peeling rate by 38%, 31%, and 41%, respectively. CLDHs can inhibit the formation of carbonyl and sulfoxide groups in asphalt binder under salt corrosion conditions, and the improvement effect exhibits a positive correlation with the calcination temperature. The addition of 600 °C-CLDHs reduces the change rates of *I_C_*_=*O*_ and *I_S_*_=*O*_ of asphalt binder after salt corrosion by 92.6% and 6.9%, respectively. Additionally, CLDHs can significantly reduce the leaching of organic components from asphalt binder: the incorporation of LDHs, 400 °C-CLDHs, 500 °C-CLDHs, and 600 °C-CLDHs decreases the TOC content of the solution after salt corrosion by 14.0%, 28.1%, 26.1%, and 39.4%, respectively.

## 5. Conclusions

In summary, CLDHs can effectively improve the salt corrosion resistance of asphalt binder. Indeed, 600 °C-CLDHs exhibit the optimal improvement effect, followed by 400 °C-CLDHs, while 500 °C-CLDHs show the least effectiveness. Based on the findings of CLDH-modified asphalt binder’s salt corrosion resistance, future research can focus on two key directions. First, systematically evaluate the performance of CLDH-modified asphalt mixtures, including high-temperature deformation resistance, fatigue cracking resistance, and salt-corrosion-aged water stability, to clarify the enhancement mechanism transfer from binder to mixture. Second, optimize the calcination temperature and dosage of CLDHs combined with performance tests and a cost–benefit analysis so as to provide quantitative support for the practical engineering application of CLDH-modified asphalt materials.

## Figures and Tables

**Table 1:** Basic properties of 70A.

Indicator	Requirement	Result	Indicator	Requirement	Result
Penetration (25 °C, 100 g, 5 s), 0.1 mm [15]	60–80	71	Penetration index [15]	−1.5–1.0	−1.06
Softening point, °C [16]	≥46	47.6	Ductility (10 °C, 5 cm/min), cm [17]	≥20	76.6
Wax content (distillation method), % [18]	≤2.0	1.6	Density (15 °C), g/cm^3^ [19]	Measured	1.016
Kinematic viscosity (60 °C), Pa·s [20]	180–240	196	Mass loss, % [21]	≤0.8	0.04

**Table 2:** Technical information of LDHs.

Parameter	Result	Parameter	Result
MgO/Al_2_O_3_	4 ± 0.2	Mass loss rate (105 °C, wt%)	≤0.5
LDHs (%)	≥99.5	Specific surface area (m^3^/g)	2.080
Bulk density (g/cm^3^)	0.34	Average particle size (μm)	5.078

**Table 3:** Chemical composition of basalt.

Composition	SiO_2_	CaO	Al_2_O_3_	Fe_2_O_3_	MgO	Others	Loss
Content (%)	45.27	7.38	14.22	12.84	9.30	9.14	1.85

**Table 4:** Technical properties of NaCl.

Indicator	Unit	Result	Indicator	Unit	Result
Physical state (60 °C)	/	Solid	Bromide	%	≤0.01
Relative molecular mass	/	58.44	Sulfate	%	≤0.002
Purity	%	>99.5	Phosphate	%	≤0.001
pH value (50 g/L, 25 °C)	/	7.00	Potassium	%	≤0.02
Water-insoluble substances	wt%	≤0.05	Magnesium	%	≤0.002
Loss on drying	%	≤0.5	Calcium	%	≤0.005
Solubility in water (25 °C)	g/100 mL	36.2	Solubility in water (60 °C)	g/100 mL	37.3

**Table 5:** Absorption bands of LDHs and CLDHs.

**Wavenumber (cm^−1^)**	**Functional Group**	**Wavenumber (cm^−1^)**	**Functional Group**
3689	Asymmetric stretching vibration of M-OH in the layered sheet	1071	In-plane bending vibration of M-OH in the layered sheet
3453	Asymmetric stretching vibration of -OH in interlayer water molecules	954	Out-of-plane bending vibration of M-OH in the layered sheet
3077	Overtone peak of -OH stretching vibration in interlayer water molecules	782	Out-of-plane bending vibration of interlayer CO_3_^2−^, or asymmetric stretching vibration of M-O in metal oxides
1635	Bending vibration of H-O-H in interlayer water molecules	684	Asymmetric stretching vibration of M-O in metal oxides
1380	Asymmetric stretching vibration of interlayer CO_3_^2−^	553	Asymmetric stretching vibration of M-O in metal oxides
1355	Asymmetric stretching vibration of interlayer CO_3_^2−^	448	In-plane bending vibration of M-O in metal oxides

**Table 6:** Particle sizes of LDHs and CLDHs.

Sample	LDHs	400 °C-CLDHs	500 °C-CLDHs	600 °C-CLDHs
Specific surface area (m^2^/g)	2.080	2.234	1.960	2.994
Volume-average particle size (μm)	5.078	4.740	4.856	4.309

**Table 7:** Kinetic fitting parameters for chloride ion adsorption by CLDHs.

Model Type	Sample	Dynamic Equation	*q_e_* (mg/g)	*k* (1/min)	R^2^
Pseudo-first-order	LDHs	$q_{t}=18.9613\left(1-e^{-0.1235t}\right)$	18.9613	0.1235	0.9522
	400 °C-CLDHs	$q_{t}=100.2320\left(1-e^{-0.2983t}\right)$	100.2320	0.2859	0.9949
	500 °C-CLDHs	$q_{t}=118.8002\left(1-e^{-0.1732t}\right)$	118.8002	0.1732	0.9647
	600 °C-CLDHs	$q_{t}=124.7822\left(1-e^{-0.1495t}\right)$	124.7822	0.1495	0.9525
Pseudo-second-order	LDHs	y = 0.0488x + 0.5249	20.4918	0.0045	0.9998
	400 °C-CLDHs	y = 0.0097x + 0.0324	103.0928	0.0029	0.9999
	500 °C-CLDHs	y = 0.0078x + 0.0721	128.2051	0.0008	0.9999
	600 °C-CLDHs	y = 0.0074x + 0.0758	135.1351	0.0007	0.9999

## Data Availability

The original contributions presented in this study are included in the article/Supplementary Material. Further inquiries can be directed to the corresponding author.

## Supplementary Materials

The following supporting information can be downloaded at: https://www.mdpi.com/article/10.3390/ma19030587/s1.
